# CesH Represses Cereulide Synthesis as an Alpha/Beta Fold Hydrolase in *Bacillus cereus*

**DOI:** 10.3390/toxins11040231

**Published:** 2019-04-21

**Authors:** Shen Tian, Hairong Xiong, Peiling Geng, Zhiming Yuan, Xiaomin Hu

**Affiliations:** 1 Key Laboratory of Special Pathogens and Biosafety, Center for Emerging Infectious Diseases, Wuhan Institute of Virology, Chinese Academy of Sciences, Wuhan 430071, China; tianshen410@hotmail.com (S.T.); heiheigpl@163.com (P.G.); 2 University of the Chinese Academy of Sciences, Beijing 100049, China; 3 College of Life Science, South-Central University for Nationalities, Wuhan 430074, China; xionghr@mail.scuec.edu.cn

**Keywords:** *Bacillus cereus*, cereulide, cesH, alpha/beta hydrolase

## Abstract

Cereulide is notorious as a heat-stable emetic toxin produced by *Bacillus cereus* and glucose is supposed to be an ingredient supporting its formation. This study showed that glucose addition benefited on cell growth and the early transcription of genes involved in substrate accumulation and toxin synthesis, but it played a negative role in the final production of cereulide. Meanwhile, a lasting enhancement of *cesH* transcription was observed with the addition of glucose. Moreover, the cereulide production in Δ*cesH* was obviously higher than that in the wild type. This indicates that CesH has a repression effect on cereulide production. Bioinformatics analysis revealed that CesH was an alpha/beta hydrolase that probably associated with the cell membrane, which was verified by subcellular localization. The esterase activity against para-nitrophenyl acetate (PNPC2) of the recombinant CesH was confirmed. Although no sign of ester bond cleavage in cereulide or valinomycin was demonstrated in in vitro assays, CesH could reverse the cereulide analogue sensitivity of *Bacillus subtilis* in vivo, by which toxin degradation was facilitated. Moreover, site directed mutations identified that the conserved catalytic triad of CesH might consist of Serine 86, Glutamate 199, and Histidine 227. These results help us to understand the regulation of cereulide production and provide clues for developing control measurements.

## 1. Introduction

Cereulide is a toxin secreted by spore-forming Gram-positive bacteria belonging to the *Bacillus cereus* group. As a cyclic dodecadepsipeptide consisting of three repetitions of [D-O-Leu-D-Ala-L-O-Val-L-Val], it is structurally related to the macrolide antibiotic valinomycin with [L-Val-L-O-Val-D-Val-L-Lac]_3_ [1]. Like valinomycin, cereulide is synthesized enzymatically via non-ribosomal peptide synthetases (NRPS), which are encoded by a 24 kb gene cluster tagged as Ces. Although *cesH* is a monocistron with its own promoter, other genetic elements (*cesTPABCD*) form an integral operon initiated by the *cesP1* promoter [2].

From the toxicology aspect, the same toxic effects on mammalian cells have been exhibited by cereulide and valinomycin, since both are potassium selective ionophores, although cereulide owns a much greater binding ability [3,4]. After being treated, potassium ions in the cell efflux following the concentration gradation and a lower level of potassium is achieved. This would directly trigger dysfunctions of solute transport, and cytoplasmic pH balance as well as other important physiological processes that depend on intracellular potassium homeostasis [5]. Meanwhile, changes in the ionic permeability enable the potassium diffusion across the inner mitochondrial membrane. Cation influx would inevitably neutralize the transmembrane electrical potential, which is established by the unequal distribution of protons, then the uncoupling of oxidative phosphorylation chains and cell apoptosis would be gradually initiated [6,7].

Additionally, the lipophilic of this toxin makes it easy for skins to be penetrated. As primary victims, the mucosal cells of the respiratory and intestinal tracts would be irritated [8]. Although risks of valinomycin have not been widely presented thus far, cereulide is notorious for its remarkable chemical stability, appears to be resistant to casual sterile procedures; neither autoclaving at 121 °C for 20 min nor proteolytic enzyme treatments have sufficiently inactivated accumulated ones [9]. As described in many cases, one of the syndromes caused by cereulide is characterized by vomiting. During the first six hours post ingestion, emesia accompanied by abdominal pain in patients could be observed, normally, this self-limiting illness would not last more than 24 h [10,11]. Nevertheless, since symptoms generated by acute cereulide poisoning are nearly identical to those caused by enterotoxins produced by *Staphylococcus aureus*, outbreaks caused by cereulide-producing *B. cereus* are likely to be misdiagnosed and underestimated [12]. In the last few years, lethal incidences of young adults have been attributed to cereulide, which is occasionally fatal for immune-deficient individuals or toddlers [13,14].

Cereulide-producing strains are usually recognized as food-poisoning pathogens, although relative isolates are rare in the environment [15,16]. Benefiting from the phenotypic variability and adhesive nature acquired once converted into endospores, emetic *B. cereus* contaminations on equipment and raw materials related to the food industry can be encountered frequently [17]. To suppress the growth of *B. cereus* during storage, many general approaches have been recommended, including the use of varieties of additives and alternatives to antibiotics [18,19], while research on developing specific control methods have been launched by confirming the preservation conditions favoring cereulide production. Thus far, the impacts of atmospheric oxygen and temperature have been fully elucidated [20,21], and the matrix of food has been proven to be another key factor affecting cereulide production, as well as the multiplication of *B. cereus* [22].

According to epidemic statistics for the past decades, among all of the food poisoning associated with cereulide ingestion, bread, rice, noodles and spaghetti have frequently been involved. It was suspected that these types of food contained some common ingredients (e.g., starch) that supported cereulide formation. Indeed, maximal cereulide production as well as culture preference has been observed in farinaceous food [23]. Surprisingly, most tested emetic *B. cereus* isolates were not able to digest starch due to the absence of related enzymatic elements [24]. It was therefore hypothesized that the promotion was due to glucose, the essential hydrolysate and energy source for bacterial carbohydrate metabolism, which needs to be clarified.

In this study, the effects of glucose supplement on cereulide production and on the transcription levels of the genetic determinants, including *cesA* and *cesH* related with non-ribosomal polypeptide synthesis, and *ilvB* (a major branched-chain amino acids (BCAAs) biosynthetic gene) related with the substrate accumulation [25], were analyzed. Furthermore, the *cesH* knockout mutant and the complemented strain were constructed and the effect of deletion on cereulide formation was surveyed. Our results demonstrated that *cesH* displayed a negative effect on cereulide production. Followed by predicting an alpha/beta hydrolase fold in the protein encoded by *cesH*, CesH was characterized as an esterase which might anchor in the cell membrane of B. cereus AH187. However, no activity against cereulide or its analogue valinomycin was demonstrated in vitro. Considering the inevitable defect of detergent usage for the enormously hydrophobic substrate, CesH was subsequently expressed in *Bacillus subtilis* 168, both the valinomycin resistance acquisition by the host and signs of toxin degradation indicated it functioned normally in vivo. Moreover, through site directed mutation, the catalytic core of CesH was identified as a combination of Serine 86, Glutamate 199, and Histidine 227.

## 2. Results

### 2.1. The Negative Effect of cesH on Cereulide Production

**Figure 1:**
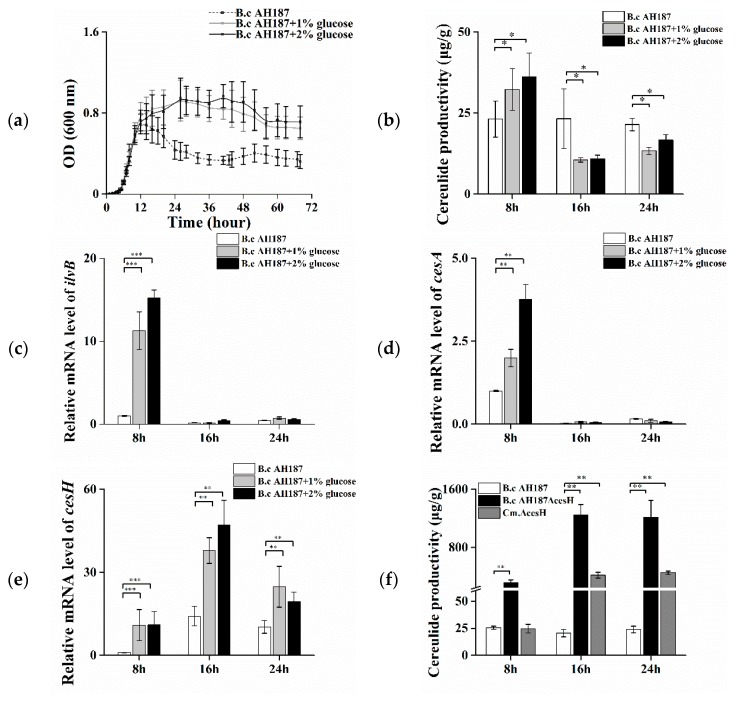
(**a**–**f**) Effects of glucose and *cesH* on the cereulide productivity of *B. cereus* AH187. (**a**) 72 h growth curve; (**b**) Cereulide productivity in CADM containing 0% (dash line), 1% (grey line), and 2% (black line) (*v*/*v*) glucose at the 8th, 16th, and 24th hour, respectively; Relative mRNA levels of (**c**) *ilvB*, (**d**) *cesA*, and (**e**) *cesH* in CADM containing 0% (white column), 1% (grey column), and 2% (black column) (*v*/*v*) glucose at the 8th, 16th, and 24th hour, respectively; (**f**) Cereulide productivity of AH187 wild type (white column), Δ*cesH* (black column), and the complementary strain Cm.ΔcesH (grey column) at the 8th, 16th, and 24th hour, respectively. Data were displayed as means and standard deviations from three biological replicates, where the significant differences are indicated by asterisks (* *p* < 0.1, ** *p* < 0.05 and *** *p* < 0.01).

The growth of *B. cereus* AH187 in CADM medium (Complete Amino acids Defined Medium) and CADM containing 1% and 2% glucose, respectively, were assessed. The effect of increasing glucose concentration was illustrated in Figure 1a, where a higher OD value and total biomass were attained from the 12th to 72nd hour with the presence of glucose, albeit with no visual difference before entry into the logarithmic phase (0–12 h). The cells at the 8th, 16th, and 24th hour, respectively, were harvested for cereulide measurement. The results showed that the addition of 1% and 2% glucose initiated a ca. 30% increase in the average amount of cereulide per biomass in the bacterial logarithmic phase, while it resulted in a ca. 30% decline when the growth entered the stationary phase (at both the 16th and 24th hour) in comparison with that in CADM without glucose (Figure 1b).

Furthermore, the transcriptional levels of genes related to cereulide production were monitored in cells at different time points. It was found that the transcription of *ilvB*, responsible for facilitating substrate accumulation, was increased 12- to 15-fold at the 8th hour, whereas it was nearly shut down at the 16th and 24th hours (Figure 1c). A similar trend was displayed on the transcriptional level of the non-ribosomal polypeptide synthetase structural gene *cesA* (Figure 1d). In contrast, the mRNA level of *cesH* was low at the early stage, but obviously increased at the latter two time points. Furthermore, it was remarkably enhanced with the addition of glucose at all three tested intervals (Figure 1e).

In addition, a *B. cereus* AH187 *cesH* null mutant (Δ*cesH*) and the complemented strain (Cm.Δ*cesH*) were constructed, respectively, and the effect of *cesH* deletion on cereulide accumulation was evaluated. At the early stage, the cereulide amount in Δ*cesH* was 12 times higher than that of the wild type (319 μg/g) but recovered to the same level with the wild type when *cesH* was complemented. At the 16th and 24th hour, the maximum production was observed in Δ*cesH* at 1250 μg/g, which was ca. 50 times higher than that in the wild type, and the complementary led to a decline in amplitude, but was still much higher than that in the wild type (Figure 1f). The data suggested that CesH displayed a negative effect on cereulide production.

### 2.2. Prediction and Characterization of CesH as a Membrane Associated Esterase

**Figure 2:**
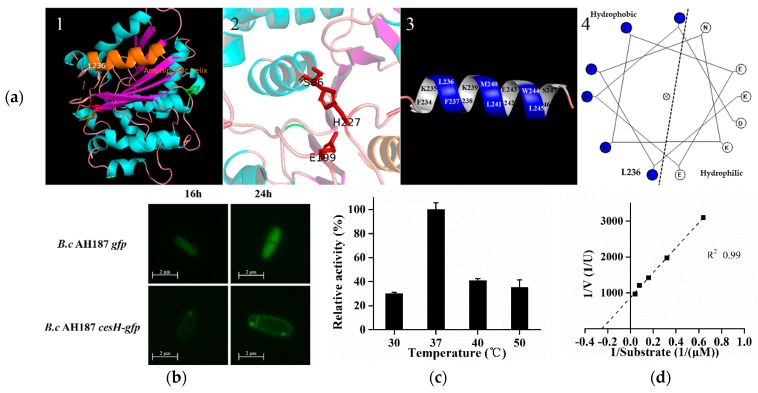
(**a**–**d**) Prediction and characterization of CesH as a membrane associated esterase. (**a**) The predicted 3D structure of CesH. 1, homologous modeling result of CesH via Phyre2.0. The alpha helix and the beta strand were separately labeled in cyan and magenta, and the yellow helix represents the amphipathic alpha at the C terminal. 2, the possible catalytic pocket. S86, E199, and H227 were labeled in Red. 3 and 4, the 3D model and helical diagram of region from 236-247, the hydrophobic residues (L236, F237, M240, L241, W244, and L245) were labeled in blue and hydrophilic residues (K235, N238, K239, E242, E243, and D246) were labeled in white; (**b**) Subcellular localization of CesH in two recombinant strains *B. cereus* AH187 *gfp* and *B. cereus* AH187 *cesH*-*gfp*. (**c**) Relative activity of CesH against PNPC2 under different temperature. (**d**) Lineweaver–Burk plot of CesH.

As predicted by the Esther database, the protein encoded by *cesH* could belong to the alpha/beta hydrolase superfamily. The secondary and tertiary details revealed via Phyre2.0 identified four alpha helices and seven beta strands in CesH. Furthermore, the 3D structure modeling analysis indicated that CesH was folded as a globular protein where S86, E199, and H277 are placed in close position, and might be involved in substrate binding and catalytic reaction. Moreover, the secondary structure element (236–247) located at the C terminal was found to be an amphipathic helix, indicating that CesH may be a membrane associated protein (Figure 2a) [26].

The *B. cereus* AH187 recombinant strains containing *gfp* alone and *cesH* tagged with *gfp* were constructed, respectively. Microscopic study showed that the fluorescence was evenly displayed within the whole cytoplasmic area when the GFP was expressed alone (*B. cereus* AH187 *gfp*), whereas it was only labeled around the entire cell wall/membrane after GPF was linked to the C terminal of CesH (*B. cereus* AH187 *cesH-gfp*). Furthermore, the heterologous expressed CesH was purified from the *Escherichia coli* BL21 (DE3)/pET28a-*cesH* strain containing Triton X-100, and its activity against para-nitrophenyl acetate (PNPC2), a candidate substrate for alpha/beta hydrolase, was detected. The activity of CesH had an optimal temperature of 37 °C (Figure 2c), a Km of 0.0396 mM and a Vmax of 92.36 U/g (Figure 2d). The data indicated that CesH could be an esterase.

### 2.3. The Effect of CesH on Cereulide Analogue–Valinomycin In Vitro and In Vivo

**Figure 3:**
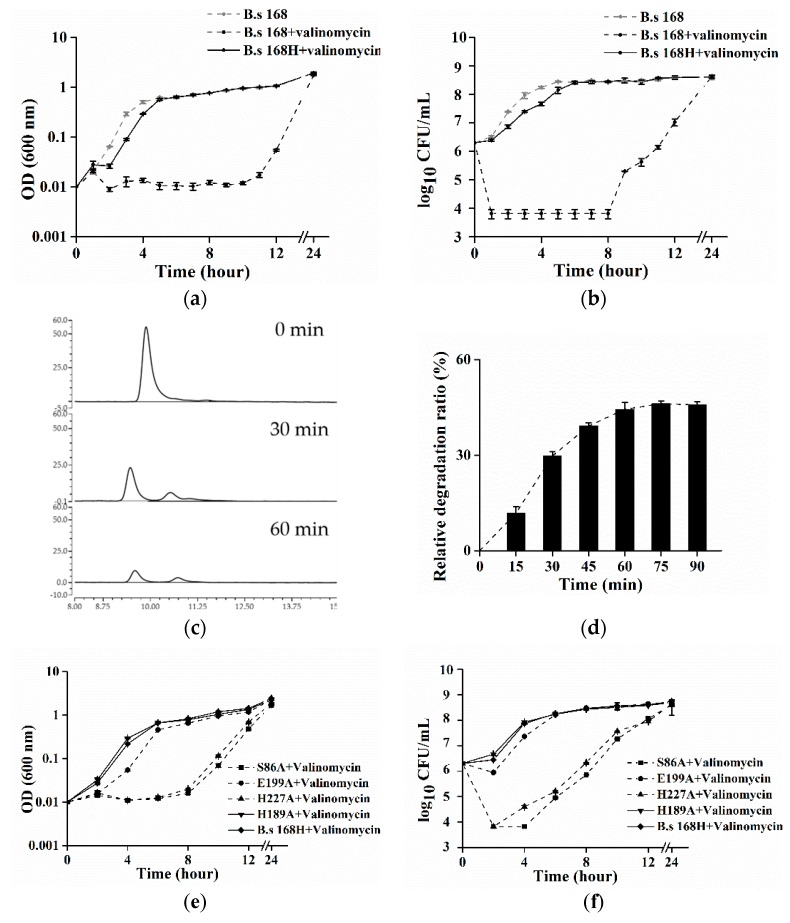
(**a**–**f**) Expression of CesH in *B. subtilis* contributed to an acquisition of valinomycin resistance in the host. (**a**,**b**) Comparison of valinomycin sensitivities of *B. subtilis* 168 (dash line) and *B. subtilis* 168H (line) by OD measuring and viable cell counting in LB medium containing 9 μM valinomycin (black), or not (grey); (**c**) chromatogram results of the reaction mixture containing valinomycin at the 0 min, 30 min, and 60 min, respectively; and (**d**) the relative degradation ratio of valinomycin in vivo counting by HPLC results, relative degradation ratio (E) at any time (t) was calculated according to the equation: E = [(Conc_168H, T = 0_–Conc_168H, T = t_) − (Conc_168, T = 0_–Conc_168, T = t_)]/Conc_168H, T = 0_ × 100%; (**e**,**f**) Proliferations and viabilities of the four mutated strains of *B. subtilis* 168H: S86A (square), H189A (down triangle), E199A (circle), H227A (up triangle) as well as the positive strain of *B. subtilis* 168H (diamond) were accessed under the valinomycin exposure condition.

Surprisingly, the purified CesH was not able to catalyze in vitro the hydrolysis of cereulide and valinomycin (data not shown). Considering that CesH is a membrane bound esterase and the toxins are extremely hydrophobic, the hydrolytic activity of the purified CesH might have been blocked, which was caused by the utilization of the detergent Triton X-100 during protein purification. As an alternative, the in vivo method was designed to evaluate the activity of CesH through assaying the bacterial resistance to valinomycin, a cereulide analogue. For this purpose, a recombinant *B. subtilis* 168 containing pHT315-*cesH* (named *B. subtilis* 168H) was constructed. The result showed that valinomycin exposure had no obvious influence on the growth of recombinant *B. subtilis* 168H containing *cesH*. While a significant negative effect on the growth of the wild type strain was detected, the viable cell number of the wild type strain dropped by nearly three orders of magnitude and OD values were fixed to a narrow range around minimum within the initiated eight hours, whereas they steadily recovered to the same level as those of the wild type strain without valinomycin and the recombinant 168H with valinomycin from the 12th hour. This suggested that the introduction of pHT315-*cesH* in vivo could neutralize the bacteriostasis effect of valinomycin (Figure 3a,b).

To determine the fate of valinomycin coexisting with *B. subtilis* 168H, the cells were incubated with 90 μM valinomycin in PBS buffer for 90 min, respectively, and the concentration of valinomycin was assayed at each 15 min. The chromatogram results of the reaction mixture at 0 min, 30 min, and 60 min were displayed in Figure 3c. Along with the incubation, the peak (retention time = 9.4 min) was split and the peak area was decreased, which was linear correlation with the concentration of valinomycin. Due to the background degradation in the wild type (ca. 7 μM/15 min), the results in *B. subtilis* 168H were presented as the relative degradation ratio. It was found that the toxin was swiftly eliminated within one hour and a maximum relative degradation ratio (ca. 45%) was kept in the remaining time.

Subsequently, to identify the key residues of CesH, site mutations were inserted into the shutter vector of *B. subtilis* 168H, where the predicted catalytic residues as well as other possible amino acids were used as candidates. It was found that the substitutions on the Serine 86 and Histidine 227 obviously impaired the valinomycin resistance of *B. subtilis* 168H, whereas that of Glutamate 199 caused a milder effect. In addition, the results on the Histidine 189 were provided as representative of other equivalent mutations (albeit at a slightly attenuated growth) (Figure 3e,f). This indicates that Serine 86, Glutamate 199, and Histidine 227 might be the necessary catalytic core of CesH.

## 3. Discussion

The original object of this study was to investigate the influence of glucose on cereulide production. It was found that the proliferation statues of strains only began to differentiate when they entered the stationary phase. Therefore, the primary changes generated by additional glucose incorporation were assessed in an expanded duration of this time point, from the late logarithmic stage (8th hour), stationary stage (16th hour), to late stationary stage (24th hour). The result at the first interval was consistent with the expectation; a higher productivity was gained with the addition of glucose. RT-PCR results revealed that this might be the direct consequence of the positive regulation on the genes participating in the non-ribosomal synthesis pathway. Even the mRNA level of *cesA* and *ilvB* were barely detected at the latter two sampling times, and the constant productivity in the control without glucose indicated that the early transcription was sufficient for the full activation of cereulide synthesis. Thus, the decreased productivity in a glucose condition at the 16th and 24th hour suggested that the existence of a negative effect was directly or indirectly caused by glucose.

Interestingly, it observed that the decreased cereulide production corresponded with the obviously higher and lasting transcription of *cesH*; both a longer culturing time and the addition of glucose could magnify this phenomenon. This most likely indicated a repression role of *cesH* on cereulide production. Furthermore, the significant difference between the wild type and Δ*cesH* strains in cereulide production was provided as phenotypical evidence for verifying the repression function of *cesH*. The deletion of *cesH* led to a dramatic increase in the cereulide yield. Meanwhile, an obvious decreased bacterial growth was observed in the mutant (Supplementary Figure S1). This might be triggered by metabolic burden due to the massive generation of cereulide, so that the substrates, which are also essential for plenty of physiological progresses, were exhausted.

According to the bioinformatics prediction, the protein encoded by *cesH* was an alpha/beta fold hydrolase, moreover, an amphipathic alpha helix was identified in the secondary structure, which may mediate the membrane association for proteins lacking transmembrane regions [27]. A GFP co-expression assay verified that CesH was bound to the membranes. Together with the cellular location of cereulide, these results reminded us of the possible interaction between them, since cereulide has been found to be mainly reserved in membrane of emetic *B. cereus* [28]. This natural physical approximate was regarded as the fundamental of substrate-enzyme recognition. Although a domain (GO: 0000287), interpreted as the magnesium-binding site, was hit in the amino acid sequence, it was found that CesH could normally hydrolysis pNPC2, a universal chromogenic substrate for lipase/esterase, without requiring a co-factor. However, our results found that the ester bond of cereulide could not be cleaved by CesH in vitro, which might be attributed to the existence of detergent during incubation, as it could prevent the binding of the enzyme by surrounding the substrate as previously indicated [29].

Tempelaars et al. showed that cereulide and valinomycin displayed an antimicrobial effect against the tested Gram-positive bacteria in alkaline circumstances, which, however, did not contain emetic *B. cereus* since it displayed less sensitivity to valinomycin and resistance to cereulide, most likely due to unidentified mechanisms [30]. As orthologs of cesH have been found in non-emetic *B. cereus* [31], the anti-bacteriostasis role of CesH was analyzed in *B. subtilis*. Our data showed that the growth of *B. subtilis* could only be inhibited by commercial valinomycin but not by extracted cereulide. As described by Tempelaars, a possible explanation is that the proton motive forces contributed by the transmembrane electrical potential of Gram-positive bacteria are only produced in alkaline circumstances, which are essential for biological events depending on the active or passive transport. When there was enough potassium in the environment, this inhibition could be relieved as both cereulide and valinomycin act as a K^+^ ionophore. Notably, the extracted cereulide was found to be rich in potassium via specific fluorescent probes (data not shown), although a desalting step was performed using a silico column. In following studies, valinomycin was treated as an alternative to cereulide since it shares a similar chemical structure, synthetic pathway and toxicology with cereulide and has often been used as an analogue in function analysis. Once the recombinant plasmid pHT315-*cesH* was transformed, a valinomycin resistance acquisition by *B. subtilis* 168 was observed in the microtiter plate (data not shown) and in the flask inoculation assays. To examine whether the mechanism of bacteriostasis neutralization effect was mediated by hydrolyzing, rather than by decreasing the affinity for valinomycin, metabolism assays of strains containing an intact *cesH* fragment were carried out and the concentration of residual toxin was determined by HPLC. It should be noted that the *B. subtilis* wild type owned an innate capacity of valinomycin consumption, which could also be speculated from the growth results, as both the OD value and the number of colonies started to rise at a certain time post valinomycin exposure. With the action of CesH, this toxin degradation was dramatically accelerated.

Finally, studies on the possible catalytic mechanism were launched by reviewing the secondary structure of CesH. Despite missing one beta strand in the computer-assisted architecture (i.e., CesH had 7 beta strands whereas the normal alpha/beta hydrolase containing 8 ones), it was able to identify CesH as an alpha/beta fold protein [32]. Since enzymes belonging to this superfamily have evolved in a divergent mode, functional cores are usually individual residues, instead of certain conserved fragments or domains. Only the combinations and locations of amino acids are invariable in the members given their diverse catalytic activities [33]. Generally, these critical residues include an amino acid with the capability of performing nucleophilic attacks on target chemical bonds, which are frequently chosen from serine, cysteine, and aspartic acid. As the active site, it is in between the two glycines located between the third helix and the fifth strand, before this fragment is finally folded into a sharp turn in the tertiary structure, called the “nucleophile elbow”. As part of the catalytic machinery, it was thought to be involved in the formation of low barrier hydrogen bonds with the acidic partner of aspartic acid or glutamate, which was next to the seventh strand [34]. The transferring proton is finally accepted by the histidine preceding the last helix. According to the sequential order of all motifs in CesH, all of the candidates, as well as the penultimate histidine, were picked and converted into alanine via site direct mutation, and identification was accomplished by recording the diversity of cereulide analogue resistance. The consequence of substitution at glutamate 197 was nearly the same as that of H189A, displaying no effect. Furthermore, the negative effect of the mutation of E199A was not obvious as those of S86A and H227A. The possible reason was hypothesized in that the work of glutamate 199 could be partly fulfilled by the adjacent glutamate 197. This is corresponding to the predicted 3D structure of CesH (Supplementary Figure S2), since S86, H199, and E227 are proximal and placed in a pocket, whereas H189 is located at the surface of the protein. However, whether the effects of the deletions are due to improper folding remains unclarified. Furthermore, confirmation of glutamate also pointed out the esterase activity of CesH, which is usually an aspartic acid in other subfamilies [35].

In summary, *cesH* could be translated into an esterase, while the activity against toxins was only demonstrated when it was expressed in vivo. Most likely similar to the mode of the antibiotic resistance gene, the role of *cesH* is to neutralize the adverse effects caused by cereulide on the ionophore-producing host itself. This property might contribute to its self-resistance and be necessary for expelling the competitive bacterium that prefers to occupy the same niches in the ecosystem [36]. From the aspect of physiological significance, this regulation shifted the equilibrium toward the side of branch amino acids. Additionally, it might be initiated once the harsh environment becomes suitable for growth, which was line with the alternation led by glucose addition. Corresponding mechanisms still need to be clarified.

## 4. Materials and Methods

### 4.1. Strains, Plasmids, Primers, and Growth Condition

The bacterial strains and plasmids used in this study were listed in Table 1. Primers used in this study were listed in Table 2. The CADM medium was prepared as described in Agata et al. [37]. Unless mentioned, the *B. cereus* strains were cultured in Luria Bertani medium (LB) at 30 °C, *E. coli* strains for DNA manipulation were grown in LB at 37 °C. The concentrations of antibiotics used in this study were 100 μg/mL for ampicillin, 10 μg/mL for kanamycin, and 5 μg/mL for erythromycin.

### 4.2. Extraction and Quantification of Cereulide via High Performance Liquid Chromatography (HPLC)

To compare the cereulide productivity of cells grown in CADM and glucose supplementary medium, the crude extraction of cereulide was prepared as follows: A total of 10 mL bacterial culture was grown for 8, 16, and 24 h, respectively, and centrifuged (12,000 *g*, 10 min) at room temperature before the pellet was weighed and resuspended in an equal volume of methanol. The solution was centrifuged (12,000 *g*, 10 min) again, and then the supernatant was blown for dryness under nitrogen, and the obtained extraction was further dissolved by 150 μL of methanol.

To obtain purified cereulide for the activity assay, the cells of 1 L bacterial culture with 16 h of growth were harvested by centrifugation (7000 *g*, 20 min) and dissolved using 200 mL methanol. The obtained crude extracts were diluted with the same volume of water and the mixture was applied to a C8 SPE column (Bojin, China) for purification. Cereulide was finally eluted with 100% methanol, and the obtained solution was concentrated to an appropriate volume using nitrogen blowing.

The HPLC assay was carried out on the Thermo Ultimate 3000 platform equipped with a Welch LP- C18 column (4.6 × 250 mm, 5-μm particle size). The components were separated with a mixture of 1‰ acetic acid and absolute methanol (5%:95%, *v*/*v*) at a flow rate of 1 mL/min, with a sample injection volume of 50 μL. The absorbance at 220 nm was monitored. The retention time of cereulide was 8.6 min and the peak area was obtained for calculating its concentration, based on the standard curve established by the valinomycin concentration gradient. Each quantification was carried out in triplicate. All of the organic solvents used in this section were of HPLC grade.

### 4.3. Bioinformatic Analysis of CesH

The sequence of CesH (A1BYH1) was obtained from UniProt [38], which was subsequently submitted to the Esther database to determine its superfamily [39]. Phyre2.0 was employed for homologous modeling where the α chain of murine hydrolase (PDB ID: 1C6R) was chosen as the backbone [40]. The possible functional residues were picked through the specific locations in the alpha/beta hydrolase (Supplementary Figure S2). The 3D structure of CesH was illustrated using PyMOL 2.2, and the diagram of the amphipathic alpha helix (236–247) at the C terminal was plotted using Helixator [41].

### 4.4. RT-PCR and DNA Manipulations

RNA was extracted using the Direct-zol RNA kit (ZYMO, USA). Transcriptions of selected genes (*cesA*, *ilvB*, and *cesH*) and the 16S rrn gene (chosen as the reference) were monitored using the primers listed in Table 2. RT-qPCR was carried out using the One Step SYBR PrimeScript RT-PCR Kit (Takara, Japan) as described in Ge et al. [42]. The results as calculated with the 2^−∆∆Ct^ method were presented as relative gene expression fold.

The *cesH* null mutation was constructed via homologues recombination. Two ca. 800 bp up- and down-stream fragments of *cesH* as well as the kanamycin resistance cassette were amplified using the primer pairs 1/2, 3/4. and 5/6. After being digested using the enzymes indicated in Table 2, these fragments were ligated into the shutter vector pHT315ts, then the recombinant plasmid (pHT315ts-*cesH::kana*) was transformed into *B. cereus* AH187 by electroporation. The screening of AH187Δ*cesH* was based on the method described previously in [43]. To obtain the complementary strain, a fragment containing the intact *cesH* and its own promoter was amplified using primer pair 7/21, the amplifier was inserted into the vector pHT315, and the resulting plasmid pHT315-*cesH* was electroporated into *B. cereus* AH187Δ*cesH*.

To assay the expression and activity of CesH in vitro, the *cesH* gene fragment was amplified using primer pair 20/21 from the genome of *B. cereus* AH187 and inserted into pET28a digested with *EcoR*I and *Hind*III. The recombinant vector was transformed into BL21 (DE3).

For subcellular localization, a *gfp* fragment was amplified from pMD18T-*kana*-*gfp* using primer pair 22/23. Equal amounts of this amplifier and the *cesH* fragment were mixed as the template, then an overlapping PCR reaction was carried out using primer pair 7/23. The extended fragment *cesH-gfp* was inserted into pHT315 to construct the *B. cereus* AH187 GFP fusion strain AH187*cesH-gfp*. Positive colonies were cultured in LB medium for 16 and 24 h, then cells were collected for observation under a phase contrast microscope (Olympus BX51, 100× objective, 460–550 nm light spectrum).

In addition, overlapping PCR techniques were also employed to construct the *cesH* site directed mutants. The procedure for constructing S86A was exemplified. The whole *cesH* sequence was divided into two parts according to the mutation site; these two segements were separately cloned using primer pairs 7/10 and 9/21. Equal amounts of two amplifiers were mixed as the template, the modified *cesH* fragment was obtained via PCR using primer pair 7 and 21 and inserted into pHT315 to prepare the *B. subtilis* S86A strain. Using a similar strategy, *B. subtilis* S86A, H189A, E197A, E199A, and H227A were also obtained.

### 4.5. Growth Measurements

To survey the effect of glucose on the growth, cells of AH187 were subcultured in 50 mL CADM and glucose supplementary medium (CADM containing 1% and 2% glucose), respectively. To determine the variety in valinomycin sensitivity, cells of *B. subtilis* 168H, S86A, H189A, E197A, E199A, H227A and the wild type *B. subtilis* 168 were cultured in 50 mL LB with the addition of 9 μM valinomycin. The initial culture density of the cells was approximately 2 × 10^6^ CFU/mL and were grown at 220 rpm and 30 °C. OD_600 nm_ values were measured at appropriate intervals. Simultaneously, 100 μL of culture at each interval was serially diluted and sprayed on the LB agar for incubation at 30 °C overnight for viable cell counting. The results were presented as log_10_ CFU/mL. Each experiment was carried out in triplicate.

### 4.6. Activity Assays of CesH

To assess the activity of CesH in vitro, the protein was induced by the addition of IPTG during the growth of *E. coli* BL21 (DE3) pET28a-*cesH* and purified by a Ni-NTA purification kit (Novagen, Darmstadt, Germany) following the manufacturer’s instructions. After being checked by SDS-PAGE, the concentration was determined using the BCA assay kit (Takara). The measurement of the esterase activity was performed as described [44]. A total of 0.88 mL 50 mM Tris-HCl buffer (pH = 8.0) containing 1% (*w*/*v*) Triton X-100 and 0.02 mL substrate solution (10 mM para-nitrophenyl acetate in HPLC grade acetonitrile) were mixed thoroughly, and the reaction was initiated by adding 0.1 mL (10 μM) purified CesH solution. In the control, the same volume of buffer was added instead. After incubation at 30 °C for 10 min, the absorbances of the mixtures at 405 nm were measured. Each assay was carried out in triplicate. One unit of enzyme was defined as the amount of enzyme to release 1 μmol para-nitrophenol per minute. The optimum temperature was acquired by accessing the activity of CesH ranging from 30 to 50 °C. Enzyme kinetic assays were performed at 37 °C, where the final concentration ranged from 1.5 to 25 μM. The parameters were calculated by plotting according to the Lineweaver-Burk equation using Origin 9.

To determine whether CesH has the ability to degrade cereulide or valinomycin in vitro, the toxin was first diluted to 20 mM using 50 mM Tris-HCl buffer (pH = 8.0) containing 1% (*w*/*v*) Triton X-100, and then mixed with an equal volume of CesH solution (10 μM). After incubation at 37 °C for 60 min, the remaining toxin was extracted with two volumes of methanol, which were subjected to direct HPLC assay.

To survey the degradation activity of CesH on the valinomycin in vivo, *B. subtilis* 168H and wild type were pre-cultured overnight. After washing with PBS buffer three times, 2 × 10^8^ CFU was resuspended in 1 mL PBS buffer containing 90 μM valinomycin. A total of 100 uL of the mixture was taken and extracted by 100 uL ethyl acetate twice, and the resulting organic solution was then blown to dryness under nitrogen, which was redissolved in 150 μL methanol for the HPLC assays. The concentration was assessed by the size of peak (retention time = 9.6 min) and the relative degradation ratio (E) at any time (t) was calculated according to the following equation:

E = [(Conc_168H, T = 0_ − Conc_168H, T = t_) − (Conc_168, T = 0_ − Conc_168, T = t_)]/Conc_168H, T = 0_ × 100% (1)

## Figures and Tables

**Table 1:** Strains and plasmids used in this study.

Strains and Plasmids	Characteristics	Reference
*B. cereus* AH187	Wild cereulide-producing isolate	Ehling-Schulz, 2005
*B. cereus* AH187 Δ*cesH*	a kanamycin gene fragment was taken the place of *cesH* in AH187; Kana^R^	This study
*B. cereus* AH187 Cm.Δ*cesH*	AH187Δ*cesH* complemented with pHT315-*cesH*; Kana^R^ Ery^R^	This study
*B. cereus* AH187 *gfp*	AH187 containing pHT315-*gfp*	This study
*B. cereus* AH187 *cesH*-*gfp*	AH187 containing pHT315-*cesH*-*gfp*	pHT315-*cesH*-*gfp*
*B. subtilis* 168	Wild isolate	Spizizen, 1958
*B. subtilis* 168H	*B. subtilis* 168 containing pHT315-*cesH* for expression; Ery^R^	This study
*B. subtilis* S86A	*B. subtilis* 168H containing an alanine substitution at serine 86 of CesH; Ery^R^	This study
*B. subtilis* H189A	*B. subtilis* 168H containing an alanine substitution at histidine 189 of CesH; Ery^R^	This study
*B. subtilis* E197A	*B. subtilis* 168H containing an alanine substitution at glutamate 197 of CesH; Ery^R^	This study
*B. subtilis* E199A	*B. subtilis* 168H containing an alanine substitution at glutamate 199 of CesH; Ery^R^	This study
*B. subtilis* H227A	*B. subtilis* 168H containing an alanine substitution at histidine 227 of CesH; Ery^R^	This study
*E. coli* BL21 (DE3) pET28a-*cesH*	*E. coli* BL21 (DE3) containing pET28a-*cesH* for protein expression	This study
pMD18T-Kana-gfp	pMD18T plasmid containing a kanamycin resistance cassette and a *gfp* gene	This study
pHT315ts-*cesH::kana*	Shutter vector containing the flank region of cesH and kanamycin resistance fragment, and a temperature sensitive replicon	This study
pHT315-*cesH*	Shutter plasmid containing the intact *cesH* fragment and its promoter	This study
pHT315-*gfp*	Shutter plasmid containing the intact *gfp* fragment and the promoter of *cesH*	This study
pHT315-*cesH*-*gfp*	Shutter plasmid containing the intact *cesH* with a *gfp* fragment fused in the c-terminal	This study
pET28a-*cesH*	Expression vector containing the complete sequence of *cesH*	This study

**Table 2:** Primers used in this study.

Order	Sequence	Features
1	GCGGGATCCGCAATCCCCCCTAGCTATG	Forward primer for cloning up stream of *cesH* (*Kpn*I)
2	GCGGGTACCTCTAACACATTCATATAGTA	Reverse primer for cloning up stream of *cesH* (*BamH*I)
3	GCGAAGCTTTATTTCAATTTCATACGGGTA	Forward primer for cloning down stream of *cesH* (*Sal*I)
4	GCGGTCGACAATTTTAGCTCTTTAGTTCC	Reverse primer for cloning down stream of *cesH* (*Hind*III)
5	CGGGGATCCAGCGAACCATTTGAGGTGATAG	Forward primer for cloning kanamycin resistance cassette (*BamH*I)
6	CGGGTCGACCTAGGTACTAAAACAATTCATCCAG	Reverse primer for cloning kanamycin resistance cassette *(SalI*)
7	GCGGGTACCCAAACAAATTAGATAAGTGGATAGAGAGACA	Forward primer for cloning promoter (*Kpn*I)
8	GCGGTTCTTCTCCTTTACTCATGCAATCCCCCCTAGCTATG	Reverse primer for gfp element overlapping pcr
9	GCGGTGGCAATAGGTTTCGCTTTAGGATCCCAAGTA	Forward primer for generating mutation at residue 86
10	GCGTACTTGGGATCCTAAAGCGAAACCTATTGCCAC	Reverse primer for generating mutation at residue 86
11	GCGCATTCAGGGAATACTCAGCTAATATATTAGTTACTGT	Forward primer for generating mutation at residue 189
12	GCGACAGTAACTAATATATTAGCTGAGTATTCCCTGAATG	Reverse primer for generating mutation at residue 189
13	GCGATATTAGTTACTGTTGGTGCTAAAGAGAAAAAAATAATG	Forward primer for generating mutation at residue 197
14	GCGCATTATTTTTTTCTCTTTAGCACCAACAGTAACTAATAT	Reverse primer for generating mutation at residue 197
15	GCGACTGTTGGTGAAAAAGCTAAAAAAATAATGAAGGAT	Forward primer for generating mutation at residue 199
16	GCGATCCTTCATTATTTTTTTAGCTTTTTCACCAACAGT	Reverse primer for generating mutation at residue 199
17	GCGATTCCTAAAATTGGTGCTGGGATACCTTTAGCA	Forward primer for generating mutation at residue 277
18	GCGTGCTAAAGGTATCCCAGCACCAATTTTAGGAAT	Reverse primer for generating mutation at residue 277
19	GCGCCCGCGCTGCCACTATTAGATACCAATTTCACTTCATC	Reverse primer for *gfp*ligation
20	GCGGAATTCATGTATTATACAGAATTTGGAACGGATC	Forward primer for cloning *cesH* (*EcoR*I)
21	GCGAAGCTTTCAACTATTAGATACCAATTTCACTTC	Reverse primer for cloning *cesH* (*Hind*III)
22	GCGCATAGCTAGGGGGGATTGCATGAGTAAAGGAGAAGAAC	Forward primer for gfp element overlapping pcr
23	GCGGTACCTTATTTGTAGAGCTCATCCATGC	Reverse primer for cloning gfp element (*Kpn*I)
24	AAGCCTGATGAATTAGTTATTG	Forward primer for *ilvB* in RT-PCR assay
25	CTGGTTGACACGATAGTAA	Reverse primer for *ilvB* in RT-PCR assay
26	GATTACGTTCGATTATTTGAAG	Forward primer for *cesA* in RT-PCR assay
27	CGTAGTGGCAATTTCGCAT	Reverse primer for *cesA* in RT-PCR assay
28	TGCTTAGTTCTTGACCTA	Forward primer for *cesH* in RT-PCR assay
29	CACAACAGACTTACCTTC	Reverse primer for *cesH* in RT-PCR assay
30	GGAGGAAGGTGGGGATGACG	Forward primer for 16s rrn in RT-PCR assay
31	ATGGTGTGACGGGCGGTGTG	Reverse primer for 16s rrn in RT-PCR assay

## Supplementary Materials

The following are available online at https://www.mdpi.com/2072-6651/11/4/231/s1, Figure S1: Growth curve of *B. cereus* AH187 and the derived strains in CADM medium. Figure S2: Location of possible functional residues.
