# Supplementary material for: CesH Represses Cereulide Synthesis as an Alpha/Beta Fold Hydrolase in Bacillus cereus
Source: Toxins (Basel). 2019 Apr 21;11(4):231. doi: 10.3390/toxins11040231 (PMC6521208; doi:10.3390/toxins11040231)
Supplement: Supplementary file 1 [file toxins-11-00231-s001.pdf]

Supplementary Materials: CesH Represses Cereulide Synthesis as An Alpha/Beta Fold Hydrolase in *Bacillus cereus*

Shen Tian, Hairong Xiong, Peiling Geng, Zhiming Yuan and Xiaomin Hu

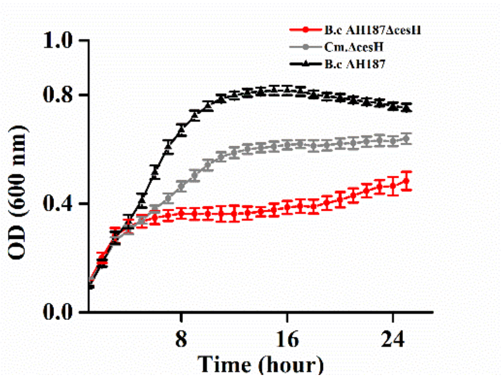

Figure S1. Growth curve of *B. cereus* AH187 and the derived strains in CADM medium. *B. cereus* AH187 (black triangles), *B. cereus* AH187 *cesH* deletion mutant (red cycle), and the complementary strain (grey cycle) were individually cultured in CADM medium for 24 h, respectively, the initial $OD_{600\ nm}$ = 0.01.

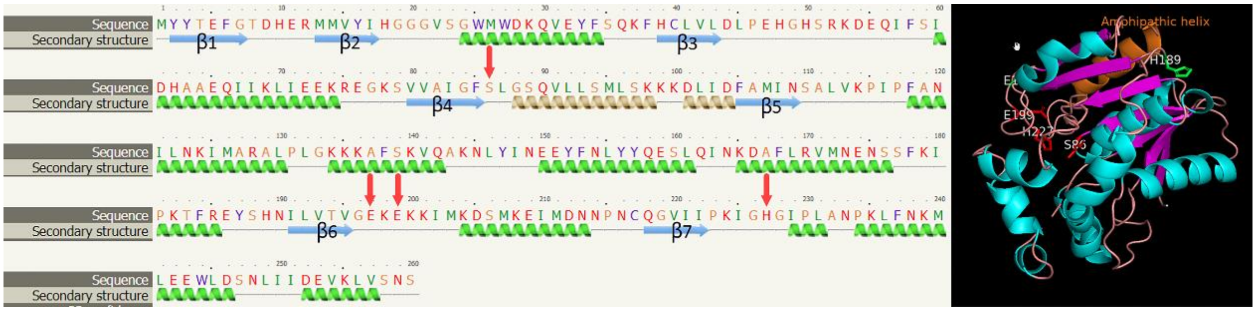

Figure S2. Location of possible functional residues. Left, the distribution of the alpha helix (green and brown) and beta strand (blue) in the secondary structure of CesH. Considering the location relationship between beta strands and the catalytic triad, the possible functional residues were searched near the predicted beta strands. The S86 locate behind the fourth beta strand and is in between the two glycines (G84 and G88). E197, and E199 were possible residues located behind the sixth strand. Behind the last beta motif, only H227 was found. Right, positions of all candidates in site mutation assays are shown on 3D structure, H189 was on the surface of the protein.
